# Morbidity of 200 Consecutive Cases of Hand-Assisted Laparoscopic Living Donor Nephrectomies: A Single-Center Experience

**DOI:** 10.1155/2012/121523

**Published:** 2012-02-22

**Authors:** Pedro W. Baron, Ramzi Ben-Youssef, Okechukwu N. Ojogho, Arputharaj Kore, D. Duane Baldwin

**Affiliations:** ^1^Transplantation Institute, Loma Linda University Medical Center, Loma Linda, CA 92354, USA; ^2^Department of Urology, Loma Linda University Medical Center, Loma Linda, CA 92354, USA

## Abstract

*Background*. Recipients of laparoscopically procured kidneys have been reported to have delayed graft function, a slower creatinine nadir, and potential significant complications. As the technique has evolved laparoscopic donor nephrectomy technique is becoming the gold standard for living donation. *Study Design*. We retrospectively reviewed the data of the first 200 hand-assisted laparoscopic living donor nephrectomies performed between January 2003 and February 2009. The initial 41 donors and their recipients (Group 1) were compared to the next 159 donors and their recipients (Group 2). The estimated blood loss, serum creatinine at discharge and 6 months, and the incidence of delayed graft function and perioperative complications were analyzed. *Results*. The median donor serum creatinine at discharge and 6 months was 1.2 mg/dL in each group. None of the laparoscopic procedures required conversion to an open procedure, and none of the donors required perioperative blood transfusion. The median recipient serum creatinine at 6 months after transplant was 1.2 mg/dL for each group. No ischemic ureteral complications related to the laparoscopic technique were seen. *Conclusions*. HALDN with meticulous surgical technique allows kidney procurement with very low morbidity and no mortality. This improved safety and decreased invasiveness from laparoscopic approach may further decrease morbidity of the procedure and increase organ donation.

## 1. Introduction

Despite attempts to increase the number of potential kidney donors, there continues to be a significant shortage of kidneys available to meet the demand for renal transplantation. The gap between the number of patients waiting for a kidney transplant and the number of patients receiving a transplant has widened over the last decade; this has resulted in a continued increase in the waiting time from listing to transplant.

At the end of 2008, 50,624 patients were active candidates waiting for kidney transplant, but only 10,551 patients received a deceased donor kidney, and 5,966 received a living donor kidney [1]. Living kidney donation is one of the most attractive approaches to correct the shortage of deceased kidneys available for transplantation.

Open living donor nephrectomy had been the standard procedure performed for kidney donation during the previous four decades. Large series have reported an estimated perioperative mortality of 0.03% and 0.2% major and 8% minor complication rates [2, 3]. In 1995, the first laparoscopic donor nephrectomy was reported by Ratner et al. [4], and in 1998 the hand-assisted variant of the laparoscopic donor nephrectomy was described by Wolf Jr. et al. [5]. The decreased donor morbidity, faster recovery, comparable patient and graft survival compared with open kidney donation have resulted in several major transplant centers adopting either a pure or hand-assisted laparoscopic technique as their procedure of choice for live kidney donation [6–10]. Despite extensive experience with laparoscopic donor nephrectomy, major series continue to report major complications including bleeding, transfusion, open conversion, kidney damage at the time of extraction, delayed graft function (DGF), ureteral complications, chylous ascites, small bowel obstruction, and incisional hernias [11–16]. The hand-assisted technique was introduced to make the entire procedure quicker and safer by having the hand in the abdominal cavity. It facilitates the procedure and allows the surgical team to act quickly in case of any complication such as acute bleeding.

The first hand-assisted laparoscopic living donor nephrectomy (HALDN) was performed at our institution by Dr. H. Roger Hadley and Dr. Herbert Ruckle in 1999. The laparoscopic living donor nephrectomy program was established in January 2003 based on the joint efforts of the Department of Urology and the Transplantation Institute. Several modifications of the conventional laparoscopic donor nephrectomy were instituted in an attempt to increase the safety of living donor nephrectomy and to enhance the early graft function in the recipient. These modifications included the use of a hand-assisted technique to shorten the learning curve and increase the margin of safety for the procedure, preoperative imaging with a 4-phase CT angiogram with maximum intensity projections (MIPS) and three-dimensional reconstructions, a technique to postpone hilar dissection until the end of the procedure, and finally the establishment of a donor team consisting of a laparoscopic transplant surgeon (PWB) and an endourologist (DDB).

 We report the outcome of the first 200 consecutive HALDN performed using this protocol, focusing on short-term kidney allograft function and donor and recipient perioperative complications.

## 2. Methods

We retrospectively reviewed the data of the first 200 consecutive donors who underwent HALDN using the established donor protocol as well as the data of their corresponding recipients between January 1, 2003 and February 28, 2009. Our surgical technique for HALDN has been previously reported [17]. The donor and recipient populations were divided into two groups. The initial 41 cases, which were the subject of a previous report [17] (Group 1), were compared to the subsequent 159 cases (Group 2).

Data collected on each patient included donor and recipient demographics, donor body mass index (BMI), number of donor renal arteries, donor and recipient serum creatinine at the time of discharge, and 6 months, length of hospital stay (LOS), estimated blood loss (EBL) and donor and recipient complications. DGF was defined as the need for hemodialysis in the first week following transplantation.

These data were analyzed using the Statistical Package for Social Sciences version 17.0 software (SPSS, Inc., Chicago, IL, USA) and Statistical Analysis Systems (SAS Institute; Cary, NC) version 9.2. Differences in nominal data were tested using chi-square or Fisher's exact tests when counts were small. Means of continuous variables were compared using an independent *t*-test. *t*-test results were verified using the Mann-Whitney nonparametric test. *P* < 0.05 was considered significant. Data are shown as a mean ± standard deviation (SD) unless otherwise indicated. One-way ANOVA and Kruskal-Wallis were used to compare the difference between the groups. Univariate analysis of variance was used to compare the serum creatinine of the recipient groups at discharge adjusting for peak PRA percentage.

## 3. Results

**Figure 1:**
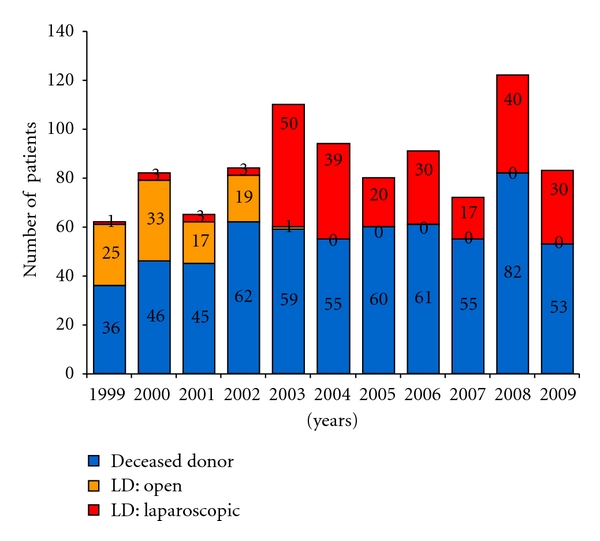
Kidney transplants by donor type and surgical technique.

From January 1, 2003 to February 28, 2009, a mean of 95 kidney transplants (a mean of 33 laparoscopic living donors and a mean of 62 deceased donors) were performed annually at Loma Linda University Medical Center (Figure 1). Following the implementation of this living donor protocol, only one donor has requested an open technique. One hundred and fifty-two nephrectomies performed during the study period were left-sided and seven were right-sided (all of these patients belong to Group 2).

The mean number of living kidney donations for the 4 years prior to the adoption of the laparoscopic technique as the procedure of choice for kidney donation was 26 per year. 35% of all kidney transplants performed per year (mean: 33 laparoscopic donors/year) during the last 6 years were living donor kidney transplants done using a hand-assisted technique. A single patient underwent open donation in Group 1, solely based upon patient preference. This patient had normal donor anatomy.

The mean age of the donor population for groups 1 and 2 is 36.4 ± 10.6 and 36.9 ± 11.9 years, respectively. More than 60% of the donors were females in both groups (Group 1: 63% and Group 2 : 62%). BMI was not significantly different between the groups (25.2 ± 5.2 and 25.6 ± 3.1 for Group 1 and 2, resp.). Over 80% of the donors in each group had a single renal artery (Group 1: 35, 85% and Group 2: 130, 82%). Thirty-one kidney donors had two renal arteries (Group 1: 12% and Group 2 : 16%). Three renal arteries were found in one donor in Group 1 and two donors in Group 2. Only one donor had 4 renal arteries (Table 1(a)). Three donors had a complete ureteral duplication (one in Group 1 and 2 in Group 2).

**Figure 2:**
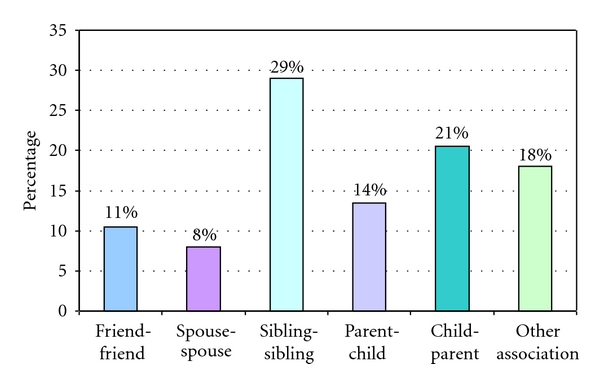
Donor/recipient relationship.

Nearly one-third of the donors (29%) were siblings of the recipients, while 21% were from children of the recipients. Eleven percent of the kidney donations came from friends of the recipients (Figure 2).

More males than females received laparoscopic living donor kidneys in each group (Group 1: 22/19 and Group 2: 94/65). The Group 2 recipients were slightly older (41.2 ± 15.3 years) than the recipients in Group 1 (39.9 ± 18.5 years), but this was not statistically significant. Thirty-four patients (21.4%) in Group 2 had a high-panel reactive antibody (PRA) greater than 20% compared to 7 patients (17.1%) in Group 1 (*P* = 0.54). There were ten recipients who had received a previous organ transplant. Eight patients received a previous kidney transplant, two patients in Group 1, and 6 patients in Group 2. One patient in Group 1 had received a heart transplant, and another one in Group 2 had received two heart transplants (Table 1(b)).

There was a significant decrease in donor median EBL in Group 2 (Group 1, 100 mL and Group 2, 50 mL, *P* < 0.0001). Donor median serum creatinine at discharge was 1.2 mg/dL for both groups (*P* = 0.55) (Table 2). Recipient median serum creatinine levels at discharge were 1.1 mg/dL (0.6–5.1) and 1.3 mg/dL (0.4–8.6) (*P* = 0.07) for Groups 1 and 2, respectively (Table 3). The donor median serum creatinine at 6 months remained stable at 1.2 mg/dL for each group, (Group 1: 1.2 mg/dL (0.8–1.9) and Group 2: 1.2 mg/dL (0.7–2.2), *P* = 0.69) (Table 2). The recipient median serum creatinine at 6 months after transplant was 1.3 mg/dL (0.8–2.2) and 1.3 mg/dL (0.4–3.3), for Groups 1 and 2, respectively (Table 3).

The median LOS for donors in Groups 1 and 2 was the same (3 days) (Table 2). For recipients, median LOS was a day less for Group 2 when compared to Group 1 (4 days versus 5 days, *P* = 0.0078, Table 3).

Fifteen kidney donors (7.5%) developed complications, three (1.5%) intraoperative, eight (4%) in the immediate postoperative period, and four (2%) in the late postoperative period (more than 6 months after donation). Seven donors of Group 1 (17%) and eight donors of Group 2 (5%) developed complications (Table 4). Four patients developed a supraumbilical incisional hernia, one at 9 months in Group 1 (0.5%) and 3 after 6 months of donation in Group 2 (1.5%). Other complications included three patients with intraoperative bleeding that did not require blood transfusion. The bleeding was controlled laparoscopically without conversion to an open procedure. The last intraoperative bleeding complication was seen in case number 59 (Group 2).

Two patients complained of significant left testicular pain (lasting longer than 3 weeks), and one patient developed a mild stricture of the external urethral meatus. One patient developed a struvite ureteral stone one week following donation and secondary acute renal insufficiency (serum creatinine peak was 4.1 mg/dL) that resolved after stent placement and subsequent stone removal (serum creatinine was 1.4 mg/dL at discharge).

Eleven of the intraoperative and perioperative complications of the donor population were classified as Grade 1 (*n* = 11, 5.5%), according to the modified Clavien classification [18] except four complications that were classified as Grade 2, 2% (four patients developed incisional hernias after 6 months of donation that required surgical repair, Group 1, *n* = 1, 0.5% and Group 2, *n* = 3, 1.5%).

There were no open conversions, bowel injuries, or readmission to the hospital for small bowel obstruction or prolonged ileus. None of the donors required blood transfusion. No complications related to multiple vessels or ureters were seen in any donor. The BMI was not significantly different between donors without and with perioperative complications.

There were a total of 25 complications (12.5%) in the transplant recipient patients of hand-assisted laparoscopic kidneys. Six recipients (15%) of Group 1 and nineteen patients (12%) of Group 2 developed postoperative complications (Table 5). A 50-year-old female developed acute allograft dysfunction on postoperative day 5, and kidney allograft biopsy showed de novo crescentic glomerulonephritis. This patient was successfully treated with plasmapheresis and cyclophosphamide. Her most recent serum creatinine was 1.3 mg/dL, almost 6 years later (2/21/09). Another 21-year-old woman with a history of focal segmental glomerulosclerosis developed a recurrent disease 5 months after transplantation. At 7 months after transplantation, her creatinine was 3.2 mg/dL but she did not require dialysis. Three patients of Group 1 and 5 patients of Group 2 developed acute rejection in the first 4 weeks after transplantation. All of them responded well to steroid I.V treatment.

One patient of Group 1 developed moderate hydronephrosis one week following transplantation. Upon revision of ureteroneocystostomy, a well-vascularized distal ureter was noted. His creatinine was 2.0 mg/dL at 3 months after transplantation. A patient of Group 2 developed thrombosis of anastomosis of the inferior polar artery to the inferior epigastric artery. He underwent revision of this anastomosis 4 hours after transplantation but developed ureteral stricture 4 months later which was treated successfully with ureteral stent and dilatation. There were no ischemic ureteral complications related to the HALDN technique.

DGF was infrequent with HALDN, only occurring in 1/41 patients (2.4%) in Group 1 and 4/159 patients (2.5%) in Group 2. A heart/kidney transplant recipient of Group 1 developed DGF due to hypotension secondary to retroperitoneal bleeding after femoral vein puncture for endomyocardial biopsy to rule out acute rejection. Four patients of Group 2 developed DGF, one due to severe hypotension after subcapsular bleeding and acute myocardial infarction, one due to acute humoral and cellular rejection, one due to acute kidney allograft ischemia secondary to a kinked renal artery anastomosis, and another one due to a renal vessel thrombosis 4 hours after transplantation. None of them resulted from the use of the laparoscopic technique.

Deaths in the perioperative period were uncommon. There were three deaths in the series, all of them in Group 2 (3/200 patients, 1.5%). Two of them died as a consequence of acute myocardial infarction, one at 9 days (he developed delayed graft function), and another one at 30 days after transplant (normal kidney transplant function). The last patient died 4 years after pancreas transplant due to rupture of an iliac artery aneurysm. His kidney transplant function was normal prior to this event. The rest of the recipients are alive, and their allografts are functioning at 6 months after transplantation (Tables 3 and 5).

## 4. Discussion

This report details our experience of the first 200 donors and recipients following the decision to implement a protocol where all patients were offered HALDN. 

Postoperative donor complication rates vary in reports from different centers. It is estimated that the total complication rate ranges from 0% to 35%, depending on how individual authors choose to define and classify major and minor complications. [18–22]. The donor complication rate of this population (major complication 5.5% and minor complication rate 2%) is comparable to other reported series. Patel et al., found a major complication rate of 4.2% and 6.8% of minor complication rate [20]. Mjøen et al. reported 2.9% of major and 18% of minor postoperative complications rated by the Clavien classification system (grade ≥ 3) [23].

 We believe that the consistent use of our hand-assisted laparoscopic living donor protocol allowed us to obtain excellent results. A meticulous and precisely dissecting laparoscopic technique, combined with the use of a detailed CT renal angiogram readily available in the operating room and evaluated preoperatively to determine the size and location of the lumbar vein branches and renal vessels, further improves the safety of the procedure and decreases the risk for bleeding complications [24]. In our experience, flipping the kidney medially facilitates the dissection of the posterior aspect of the renal artery and the lumbar vein branches. This improved exposure avoids any accidental tearing and the potential for significant bleeding which in other series has resulted in open conversion [25]. The renal artery dissection was performed only at the origin of the renal artery from the abdominal aorta and just before kidney removal in order to minimize the time in vasospasm and subsequent ischemia to the kidney. The renal vessels were transected after ligation with double hem-o-lock clips until recently, when we began routinely using a vascular stapling ligation. The EBL was significantly different among the groups having the lowest amount of EBL in the last 159 patients (Group 2, *P* < 0.0001). Our findings are similar to others including the results of the meta-analysis done by Kokkinos et al. that showed that the hand-assisted group had significantly less intraoperative blood loss than the standard laparoscopic group [26].

 Since the introduction of the HALDN technique, many studies have compared the outcome of the standard laparoscopic technique to the outcome of the hand-assisted technique, but unfortunately all resulted in equivocal findings. Kokkinos et al. compared both techniques using meta-analytical techniques. They found that the hand-assisted technique appeared to have the same donor and recipient complication rate than the standard technique but with shorter operative and warm ischemia time as well as decreased intraoperative bleeding [26].

The data from this study supports the ease and safety of the hand-assisted technique. Our experience with this technique is similar to others showing a moderate learning curve. The technique can be mastered by a surgeon with experience in advanced laparoscopic surgical procedures [27]. The successful implementation of a laparoscopic donor nephrectomy program also ultimately depends upon the surgical experience of the transplant surgery team [28–30].

In conclusion, these data showed that HALDN is very well tolerated with acceptable donor morbidity and excellent short-term allograft function. By potentially increasing the safety of the procedure while maintaining the benefits of the minimally invasive approach, HALDN may effectively increase the donor pool and may ultimately decrease the alarming gap between the donor and recipient populations.

## Figures and Tables

**Table:** (a)

Donors	Group 1 (*n* = 41) *n* (%)	Group 2 (*n* = 159) *n* (%)	*P* value
Age (years)	36.4 ± 10.6	36.9 ± 11.9	0.79
Gender			0.83*
Male	15 (37)	61 (38)	
Female	26 (63)	98 (62)	
BMI (kg/m^2^)	25.2 ± 5.2	25.6 ± 3.1	0.91**
Renal artery number			0.72***
1	35 (85)	130 (82)	
2	5 (12)	26 (16)	
3	1 (2)	2 (1)	
4	0 (0)	1 (0.6)	

**Table:** (b)

Recipients	Group 1 (*n* = 41) *n* (%)	Group 2 (*n* = 159) *n* (%)	*P* value
Age (years)	39.9 ± 18.5	41.2 ± 15.3	0.70
Gender			0.53*
Male	22 (54)	94 (59)	
Female	19 (46)	65 (41)	
BMI (kg/m^2^)	26.5 ± 5.3	26.1 ± 4.6	0.77
Peak PRA (%)			0.54*
<20%	34 (82.9)	125 (78.6)	
≥20%	7 (17.1)	34 (21.4)	
Previous transplants			0.52
None	39 (95)	153 (96)	
One	2 (5)	6 (4)	
Donor			0.14*
Living related donor	33 (80)	110 (69)	
Living unrelated donor	8 (20)	49 (31)	

**Table 2:** Donor results.

Donors	Group 1 (*n* = 41) median (range)	Group 2 (*n* = 159) median (range)	*P* value
EBL (mL)	100 (25–400)	50 (5–300)	<0.0001^∗∗+^
LOS (days)	3 (2–4)	3 (1–5)	0.03^∗∗+^
Serum creatinine at discharge (mg/dL)	1.2 (0.9–1.9)	1.2 (0.6–2.2)	0.55**
Serum creatinine at 6 months (mg/dL)	1.2 (0.8–1.9)	1.2 (0.7–2.2)	0.69**

**Refers to *P*-values based upon the Mann-Whitney test, as the data did not fit into normal distributions following the use of transformations.

^+^Refers to statistical significance at the 0.05 level.

**Table 3:** Recipient results.

Recipients	Group 1 (*n* = 41) median (range)	Group 2 (*n* = 159) median (range)	*P* value
EBL (mL)	100 (25–300)	75 (25–1000)	0.06**
LOS (days)	5 (4–18)	4 (3–32)	0.008**
Serum creatinine at discharge (mg/dL)	1.1 (0.6–5.1)	1.3 (0.4–8.6)	0.07**
Serum creatinine at 6 months (mg/dL)	1.3 (0.8–2.2)	1.3 (0.4–3.3)	0.87**

**Refers to *P* values based upon the Mann-Whitney test, as the data did not fit into normal distributions following the use of transformations.

**Table 4:** Donor complications.

Donor complications	Group 1 (*n* = 41)	Modified Clavien classification grade*	Group 2 (*n* = 159)	Modified Clavien classification grade*
Supraumbilical incisional hernia	1	2 (0.5%)	3	2 (1.5%)
Wound infection	0	1	1	1
Left testicular pain for >3 weeks	1	1	1	1
Urinary retention	1	1	0	1
Superficial gluteal thermal injury	1	1	0	1
Mild external meatus stricture	0	1	1	1
Bleeding not requiring transfusion	2	1	1	1
Ureteral stone	0	1	1	1
Readmission for fever of unknown etiology	1	1	0	1

*Kocak et al. [18].

**Table 5:** Recipient complications.

Recipient complications	Group 1 (*n* = 41)	Group 2 (*n* = 159)
Delayed Graft Function	1	4
Acute Rejection (96 Banff criteria) at 4 weeks	3 (IIA, IIA, and IA)	5 (IA, 3 suspicious, and 1 no biopsy)
Acute myocardial infarction	1	4
Urine leak at 2 weeks	0	1
Ureteral stenosis/stricture (at 1 week, 4 months)	1	1
Renal vessel thrombosis	0	1
Acute thrombosis of inferior polar artery	0	1
Vascular kinked anastomosis	0	1
Death	0	3*

*Two patients died with a functioning kidney allograft.
